# Life Cycle Assessment of Glass Fibre Recovery from Waste Composites Using Pressolysis

**DOI:** 10.1007/s12649-025-03345-6

**Published:** 2025-10-23

**Authors:** Daniel Paul, Hesam Badri, Sadik Omairey, Nithin Jayasree, James Cosby, Joe Penhaul Smith

**Affiliations:** 1 Brunel Composites Centre, College of Engineering, Design and Physical Sciences, Brunel University London, London, UK; 2 B&M Longworth (Edgworth) Ltd, Sett End Road North, Blackburn, BB1 2QG UK

**Keywords:** Composite recycling, Pressolysis, DEECOM®, Life cycle assessment, Sustainability, Industrial scale-up

## Abstract

The increasing demand for sustainable solutions in composite recycling has driven the development of novel methodologies to minimise environmental impact. This study evaluates the life cycle assessment (LCA) of the pressolysis process for recycling glass fibres from waste composites, particularly from wind turbine blades. The pressolysis process called DEECOM®, initially developed for polymer filter cleaning, utilises repeated cycles of compression and decompression to separate fibres from polymer matrices.

Three scenarios are assessed: a batch process, an improved semi-continuous process with heat recovery, and a scaled-up semi-continuous process, targeting different schemes of scale-up. Results indicate that scaling up the pressolysis process significantly reduces environmental impact, with Global Warming Potential (GWP) dropping from 95 kg CO₂ eq/kg recovered fibre in the baseline laboratory scale batch process to 2 kg CO₂ eq/kg recovered fibre in the scaled-up scenario where component capacities are increased. This represents an approximate 98% reduction per kg fibre recovered in the scaled-up scenario compared to the baseline, with the most substantial improvement achieved by increasing equipment capacity rather than the number of runs or components. The environmental impact per kilogram of recovered fibre in the best scaled-up scenario studied (2.07 kg CO₂ eq/kg) approaches that of virgin glass fibre production and landfilling (1.72 kg CO₂ eq/kg), pyrolysis (1.52 kg CO₂ eq/kg) and solvolysis (1.92 kg CO₂ eq/kg), highlighting pressolysis as a promising recycling solution.

This study demonstrates the viability of pressolysis in facilitating a circular economy by reducing reliance on landfilling and virgin raw materials, contributing to a more sustainable composite material lifecycle.

**Graphical Abstract:**

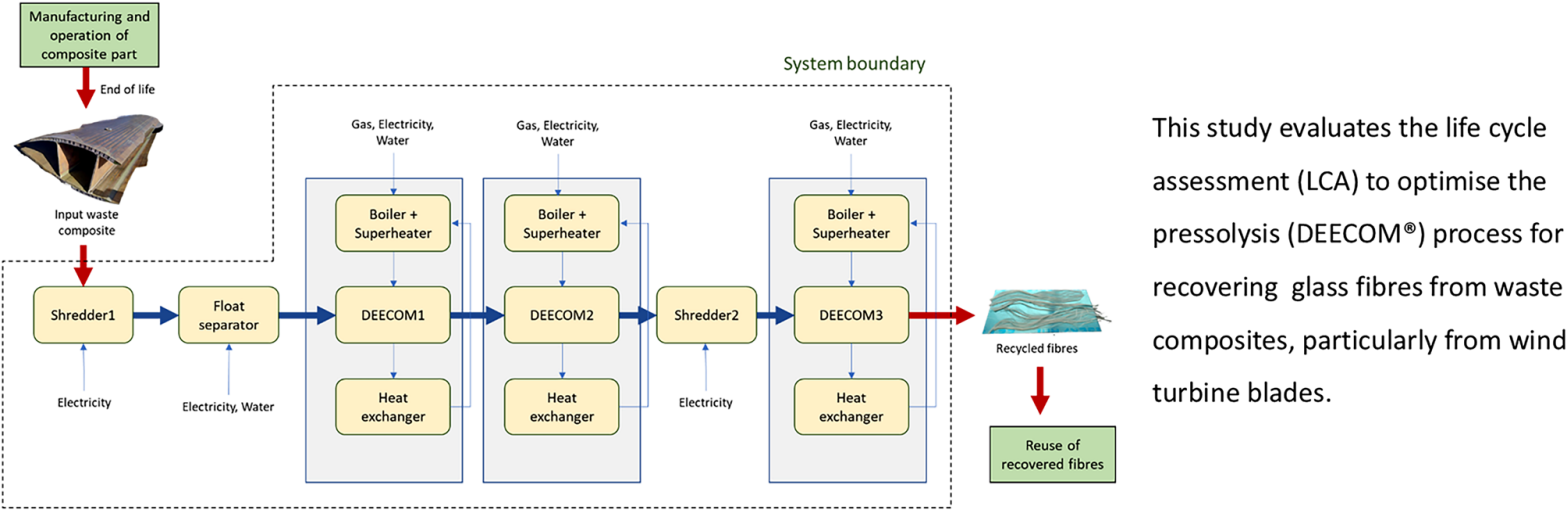

Anti-termite and antifungal activities assessments of pyrolysis tars from five Tunisians softwood and their relationships with their chemical composition analyzed by Gas-Chromatography.

## Statement of Novelty

Recovery of the constituent materials from thermoset-based fibre composites paves the way for a circular economy in various areas of manufacturing and engineering. Thermal and chemical methods used to recover the constituents, such as pyrolysis and solvolysis, often cause thermal degradation or chemical alteration of the recovered fibres, reducing their strength and surface quality and making them less lucrative for reuse in high-performance applications. In contrast, the pressolysis process (DEECOM®) employs cyclic compression–decompression of superheated steam, enabling recovery of intact glass fibres without damage, preserving their mechanical integrity for reuse. This study shows the environmental and sustainability benefits of a pressolysis process called DEECOM® which enables the fibres to be recovered without damage from end-of-life composite parts. Possible scale-up methods are also analysed, and their benefits are quantified using life cycle assessment (LCA). The results show the benefits of the current method over existing methods. The analysis aids in scaling up the process to industrial scales from the current laboratory scales.

## Introduction

In light of climate change and increasing awareness about its causes and implications, taking concrete efforts to mitigate climate change and reduce environmental harm has become essential. The United Nations (UN) has outlined 17 Sustainable Development Goals (SDGs) as a comprehensive roadmap to ensure a sustainable future [1]. There is a growing need to adopt cleaner, more circular approaches to manufacturing and disposal. One of the key strategies is captured in the “Reduce, reuse, repair and recycle” framework [1, 2]. These principles have been increasingly applied to high-performance materials like fibre-reinforced polymer composites, which are used extensively across various industries, including aerospace, automotive, construction, and renewable energy, due to their high strength-to-weight ratio, chemical resistance, and durability [3]. Their use helps create efficient structures through their superior mechanical and durability properties, and reduces operational energy through their contribution to weight reduction, hence, supporting climate goals and emissions reduction [4]. However, these same properties, particularly when involving thermoset matrices, make composites difficult to recycle, posing a major challenge for sustainable materials use.

**Fig. 1:**
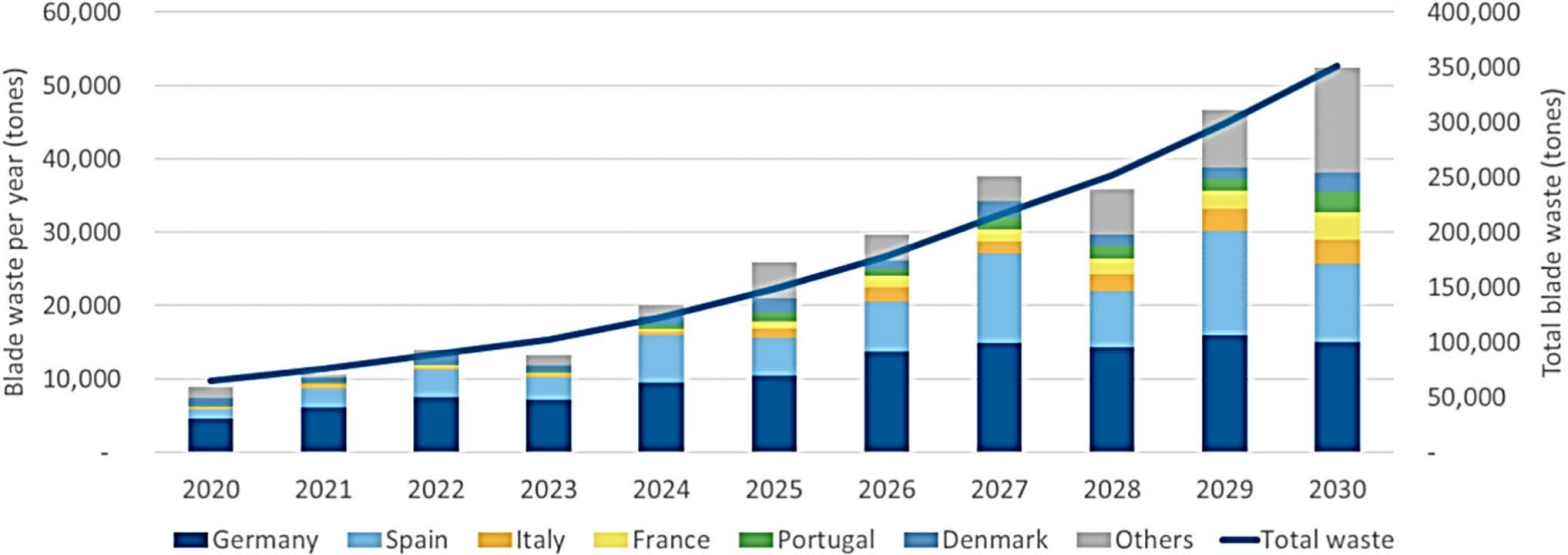
Estimated weight of decommissioned blades in Europe by 2030 (6)

This issue is especially pressing in the wind energy sector. Wind turbine blades are typically made from thermoset glass fibre composites, offering the mechanical strength and fatigue resistance needed for long service lives in harsh environments [5]. As the global wind fleet matures, an increasing number of blades are reaching their end-of-life (EoL). In Europe alone, WindEurope estimates that a total of 350,000 tons of blade from the onshore industry will reach their EoL and require disposal by 2030 [6] see Fig. 1. Historically, these blades have been disposed of through landfilling or incineration [7], practices that are increasingly restricted due to environmental regulations and sustainability concerns.

Failure to establish viable recycling routes for composite waste has serious consequences, without scalable recycling technologies, the growing stockpile of EoL composites could undermine circular economy goals and lead to substantial emissions from disposal activities [8]. Moreover, the landfilling of these durable, non-biodegradable materials represents a long-term environmental burden and a loss of embedded energy and resources.

Recycling of composites therefore serves a dual purpose:

- To recover valuable fibres and, where possible, resins from existing structures reaching end of life; and
- To inform the design of future composites with enhanced recyclability.

The positive impacts of recycling have been widely studied [9], and several technological avenues are being explored. Mechanical recycling, pyrolysis, solvolysis, and hybrid techniques each offer distinct advantages and limitations in terms of energy use, material quality, and environmental footprint [10, 11]. While some methods have reached pilot or industrial scale, many are still at lower technology readiness levels and face scale-up challenges. Despite this, ongoing research into advanced processes, such as pressolysis, offers promising routes for recovering high-quality glass fibres with reduced environmental impact.

**Fig. 2:**
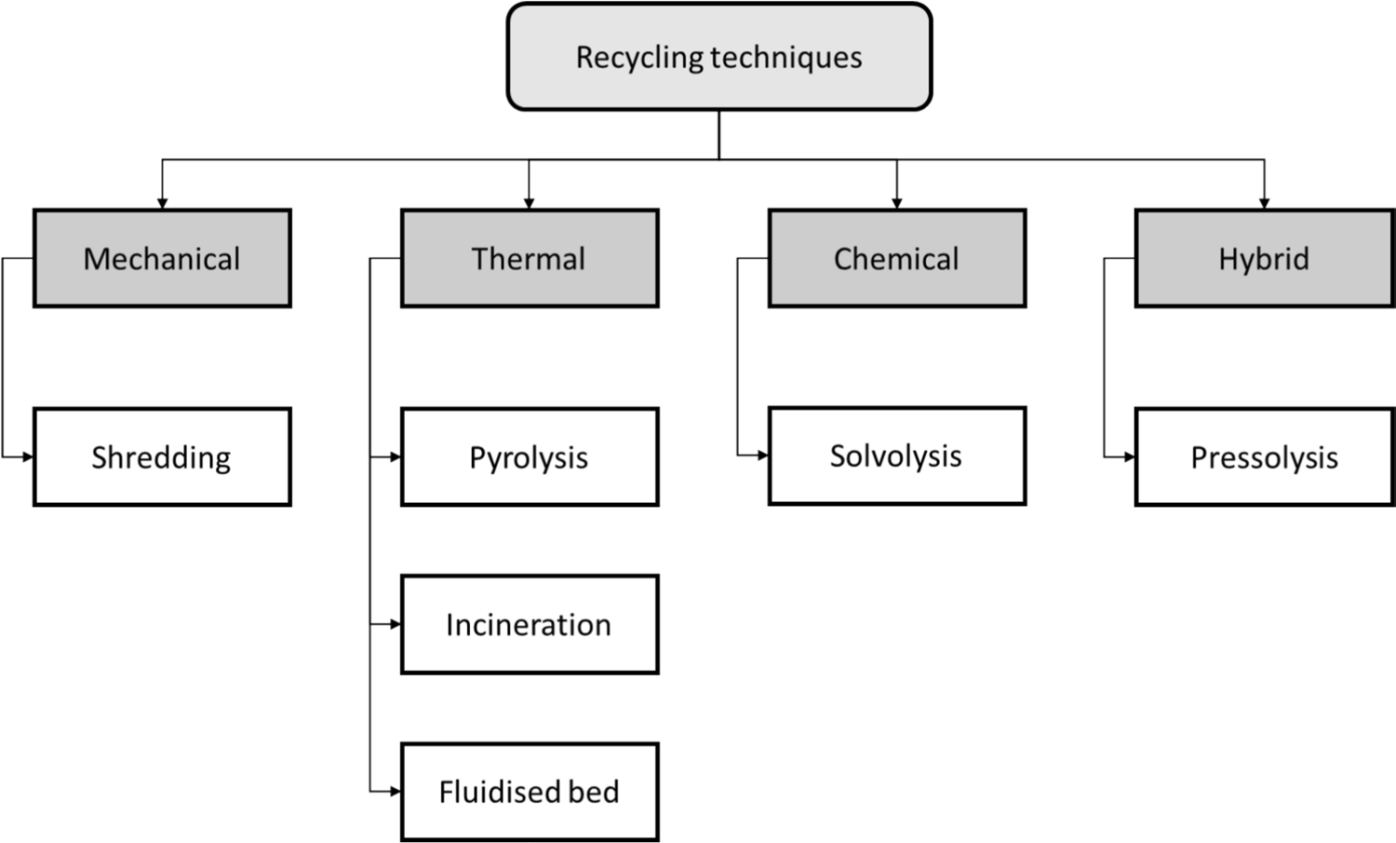
Various potential methods which can help in recycling constituents from waste composites

Examples of various recycling and material disintegration methods currently under development or use are shown in Fig. 2. As composite use continues to expand, especially in clean energy applications, scalable, low-impact recycling solutions will be essential to close the loop and align with global sustainability objectives.

While the recovery of the fibres and the polymer matrix from waste composite structures is in itself, a challenge, ensuring that the recovered material has properties suitable for further use is an additional task. Mechanical recycling is cost-effective but results in downsizing of the matrix material into smaller particles and continuous fibres into short fibres, both of which retain a surface coating of the composite resin. This can limit reusability to non-structural uses [12]. Similarly, thermal methods, such as pyrolysis have been demonstrated to degrade the mechanical performance of fibres [13].

Another challenge is the scalability of the process to industrial scales. Although many thermal and chemical recycling methods have been tested at laboratory conditions and a some have been tested at pilot or industrial scales, very few have been scaled up to commercial scales. There have been efforts to relate the technology readiness level (TRL) and the waste management score of different recycling methods. The waste management score represents how sustainable a technology is and takes into account factors such as level of material and energy recovery and environmental impact [14, 15]. Khalid et al. [16] report that the higher TRL recycling techniques in use commercially, such as mechanical recycling and pyrolysis have low or low/medium waste management score whereas medium TRL techniques such as solvolysis and fluidised bed methods have high or medium/high waste management scores. Newly developed technologies with high waste management scores but are at lab scale have even lower TRL and need to be scaled up to pilot and commercial scales.

Superheated steam, at lab scale, has been used to recover the constituents from carbon fibre-reinforced composites [17]. Pressolysis is a recently developed recycling technique [18] that employs repeated cycles of compression and decompression and superheated steam to recover the constituents from polymer-based waste composites. Initially developed to aid cleaning of polymer filters, pressolysis can also be used to recover fibres from waste composites and is gaining popularity in several industries. An example of the application of pressolysis is the recycling of carbon fibres from end-of-life sails [19]. Efforts to standardise pressolysis as a recycling method different from existing mechanical and chemical recycling processes have also been undertaken [20]. However, similar to other recycling methods, scaling up this process to commercial scales becomes necessary to extract the full potential of this technique.

The scale-up and commercialisation of several industrial processes are well-documented and studied in the past decades. Processes based on solvolysis [21], pyrolysis [22] and steam explosion [23] have been scaled-up from batch to pilot and industrial scales. This was achieved using methods such as optimising the equipment and subprocesses involved or moving from slow single-chamber method to using a continuous process. The scaled-up process has better yield and is faster. A number of studies have been conducted on the end-of-life treatment of composite wind turbine blades by methods such as landfilling, incineration, mechanical recycling, pyrolysis, solvolysis [24, 25], but has not been applied to pressolysis to the best of the authors’ knowledge.

The current study focuses on the life cycle assessment of the existing pressolysis batch-scale process and its analysis. Various relevant environmental impact parameters are considered. A pilot-scale multi-stage semi-continuous process is then proposed which, together with a heat recovery methodology, reduces the environmental impact compared to the batch scale and improves the output of the process. Finally, an economies of scale model is used to estimate the performance of a potential scaled-up pressolysis process. Comparisons with the environmental impacts of existing end-of-life methods have been performed, wherever needed, to quantify the benefits of the pressolysis process.

## Methodology of the Life Cycle Assessment

To study the environmental impacts of the reclaimed glass fibres from pressolysis, a life cycle assessment was performed focusing on the DEECOM process from the existing batch process to up-scaled semi and continuance process. The life cycle assessment (LCA) aims to provide an understanding on the environmental and economic impacts of recycling glass fibre using the pressolysis process, recognition of the benefits of the recycling process over virgin glass fibre manufacturing and other recycling methods and identification of the opportunities to improve the recycling process and enhancing the sustainability of the industry.

The LCA analysis was performed following the ISO 14040 [26] and ISO 14044 [27]. According to these standards, there are four stages in the development of an LCA model: goal and scope definition, inventory analysis, impact assessment and interpretation.

### Goal and Scope Definition

According to the ISO 14040 standard, the goal and scope definition section are important parts of an LCA methodology that define the purpose, boundaries, assumptions, exemptions considered for the model. The goal of this study is to analyse the environmental impacts of recycling glass fibre using pressolysis process and identification of opportunities to improve the process.

The system boundaries define all the processes, operations, inputs and outputs that need to be considered and integrated into the model. To define this system boundary the following assumptions and exceptions were considered:

- The reclaimed fibre is recycled from the turbine blades that were intended for landfilling.
- The model is a gate-to-gate assessment and applies averaged values rather than conducting a stochastic analysis.
- The transportation of the turbine blades to recycling facilities is not considered.
- The LCA excludes the construction of the building and manufacturing of the equipment because of their long life and negligible environmental impacts on a single process.
- Further processing (such as sizing, cutting to length and forming) of the fibres after reclaiming is not considered in the model.
- The functional unit for each scenario that is for the purpose of comparison between the scenarios is 1 kg of fibre recovered.

### Life Cycle Inventory

The life cycle inventory (LCI) of LCA involves quantification of all the inputs, outputs and processes identified within the system boundary. To quantify the system, set of data with respect to the functional unit is required to be assigned to each input, output and processes. All the data used in this study were measured to increase the accuracy of the system. The LCI for each scenario is discussed in the corresponding subsections in Section LCA models and analysis.

### Life Cycle Impact Assessment

Life cycle impact assessment is the third stage of LCA framework presented by ISO 14040 and it is used to evaluate the environmental impacts of the LCA model developed within the inventory analysis. In this study, the *LCA for Experts* software [28] is used to build and perform the LCA analysis for the pressolysis recycling process. This software has several built-in impact assessment and calculation methods and the ReCiPe 2016 methodology is used in this study. ReCiPe 2016 provides a comprehensive method to convert the life cycle inventories in any study into several life cycle impact scores which can be used analyse the environmental impact of the various stages of any process [29].

## LCA Models and Analysis

The *LCA for Experts* software was used to perform the life cycle assessment (LCA) in this study. A gate-to-gate analysis is performed to study the pressolysis process. Using the discussed LCA methodologies, 3 scenarios were analysed which are summarised in Table 1 together with their respective process characteristics, output scales, and distinguishing features. These scenarios, their life cycle inventory and corresponding models in the software are discussed in detail in the following subsections.

**Table 1:** Summary of the various scenarios studied in this paper

Scenario #	Description	Recovered fibre quantity (kg)	Key feature
1	Batch pressolysis	1.29	Laboratory scale single-stage batch unit
2	Semi-continuous with heat recovery	33	3 stages plus heat recovery
3a	Multi-run process	66, 132, 264	Multiple runs on same equipment
3b	Multi-unit process	66, 132, 264	Simultaneous runs in multiple equipment
3c	High-capacity equipment	66, 132, 264	Upsizes unit capacity

^a^The figures reported are for the recovery of carbon fibre rather than glass fibre from waste composites consisting of a matrix that may be different from the study conducted. However, they are reported here for comparison of the current process with existing conventional fibre recovery methods

### *Scenario 1:* Life Cycle Analysis of Pressolysis Batch Process (DEECOM)

**Fig. 3:**
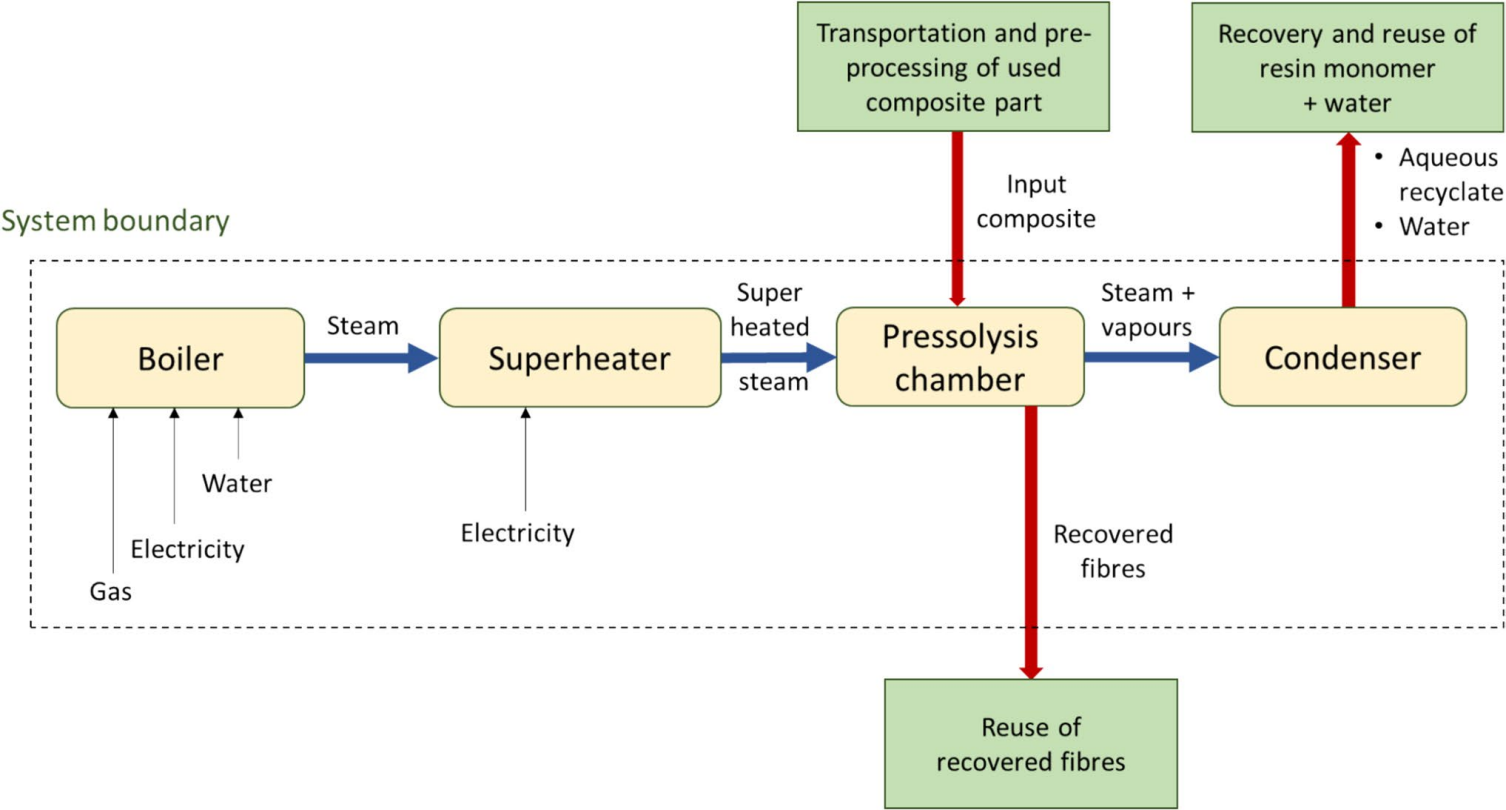
Schematic presentation of the system boundary of the pressolysis batch process (Scenario 1)

**Fig. 4:**
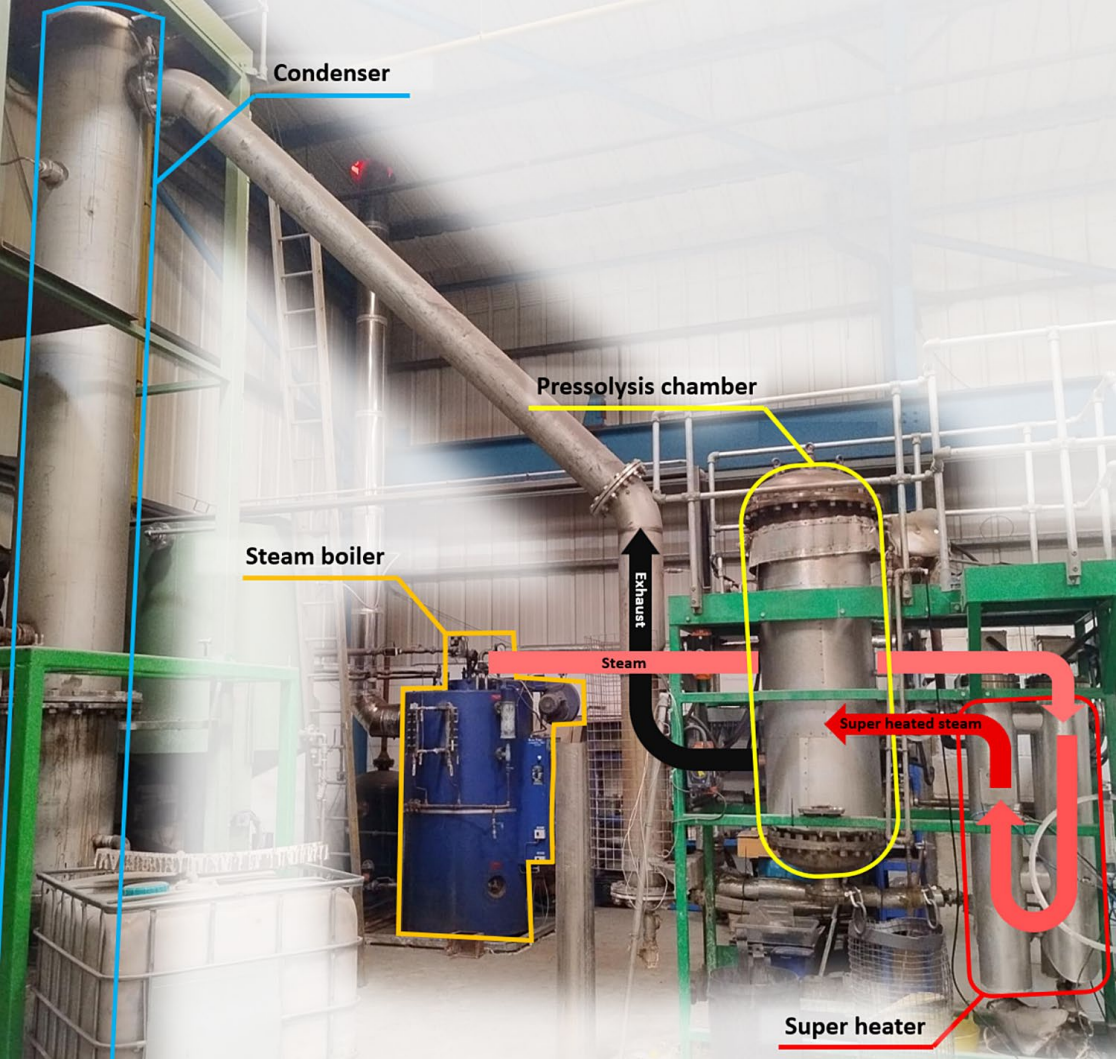
The batch-scale pressolysis processing unit at B&M Longworth showing the components and steam flow

**Fig. 5:**
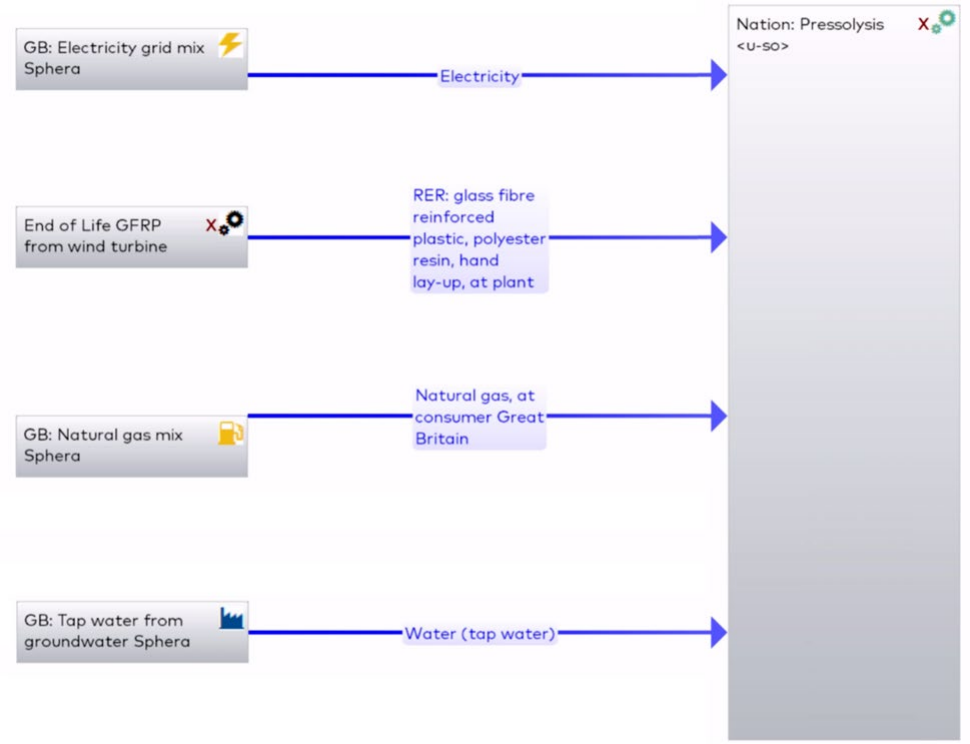
The LCA model in LCA for Experts for the batch process (Scenario 1)

The first pressolysis (DEECOM) process studied was a laboratory scale batch process which has already been constructed physically and used to collect data. The process diagram and system boundary for the process is given in Fig. 3. The process takes as input waste composite from wind turbine blades and recovers the fibres. This is achieved by using a pressolysis chamber, together with a boiler and superheater, to recover the fibres. A photograph of the operational batch-scale pressolysis unit is shown in Fig. 4. The life cycle inventory for the batch process, which lists the energy inputs and their quantities, is given in Table 2. Figure 5 shows the LCA model developed in *LCA for Experts* to perform the LCA analysis. Once the analysis is completed, the software provides various environmental impact parameters for the process. The LCA model presented here processes 3.12 kg of waste glass fibre-reinforced composite to reclaim 1.29 kg of glass fibre.

**Table 2:** Life cycle inventory for the batch process (Scenario 1)

Item	Value
Input: Composite waste from wind turbine blades	3.12 kg
Electricity	334 kWh
Natural gas	123 m^3^
Water	1430 kg
Output: Recovered fibres	1.29 kg

### *Scenario 2:* Improvement of the Process Using Improved Process and Heat Recovery

**Fig. 6:**
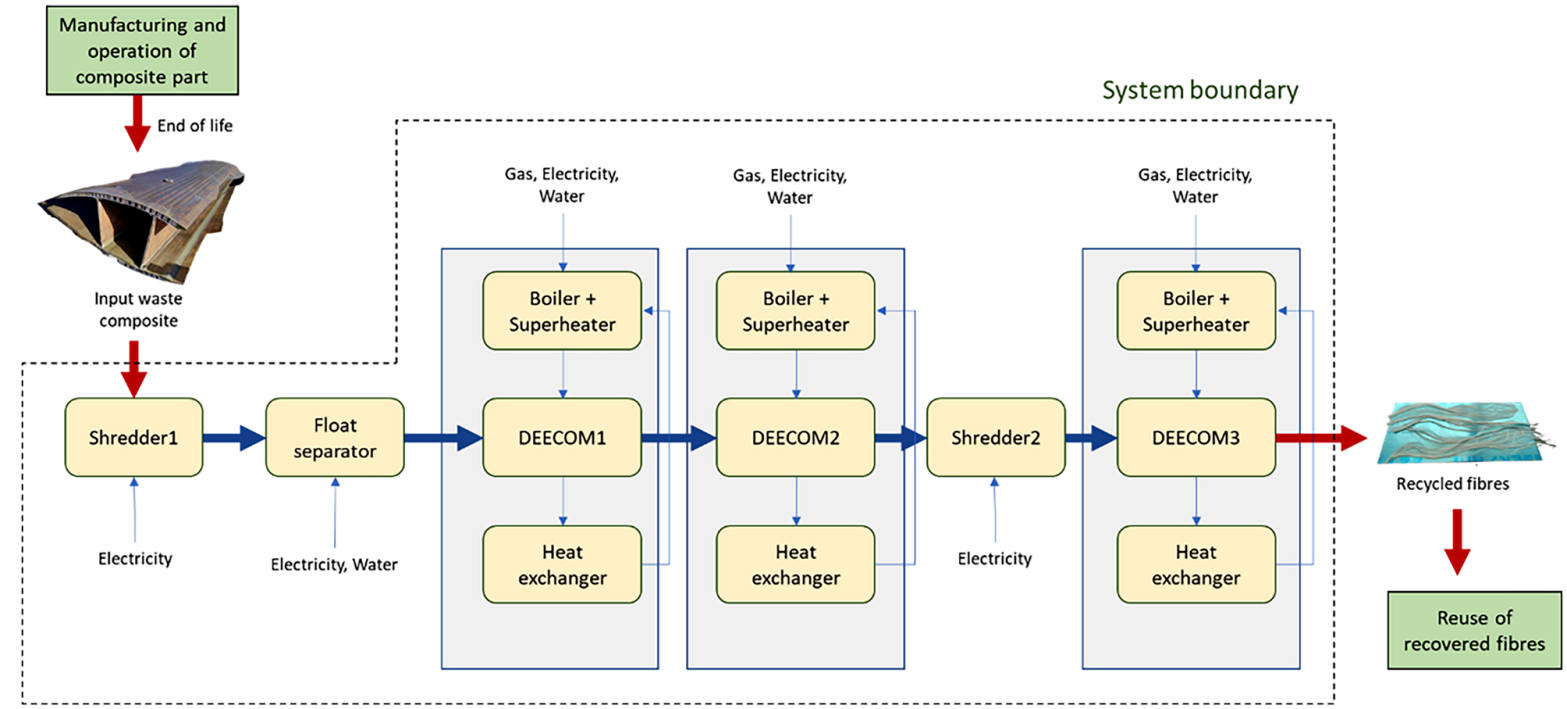
A schematic of the improved pressolysis process (Scenario 2)

A method to improve the scalability of the pressolysis process was explored by making changes to the single-stage process and reusing the heat wasted at the end of each stage. A process chart of this semi-continuous process is shown in Fig. 6. It consists of 3 stages of the pressolysis process, each of which uses separate chambers and runs at a specific temperature for a specific duration to decompose different parts of the waste composite part. These are hereafter referred to as DEEECOM 1, DEECOM 2 and DEECOM 3. Additionally, shredders and a float separator are also used. The maximum capacity of material that each component can handle is taken to be 100 kg. In each individual pressolysis stage, the superheated steam is condensed in a heat exchanger and the recovered heat is used to heat the water in the boiler. This reduces the input natural gas content needed at each stage. It is estimated that up to 60% of the energy required in the process can be recovered which is extracted during the desuperheating and condensation of the outlet steam and utilised to preheat the boiler water. Therefore, a case with 60% heat recovery is studied. This process recovers 33 kg of glass fibres from every 100 kg of waste composite processed.

**Fig. 7:**
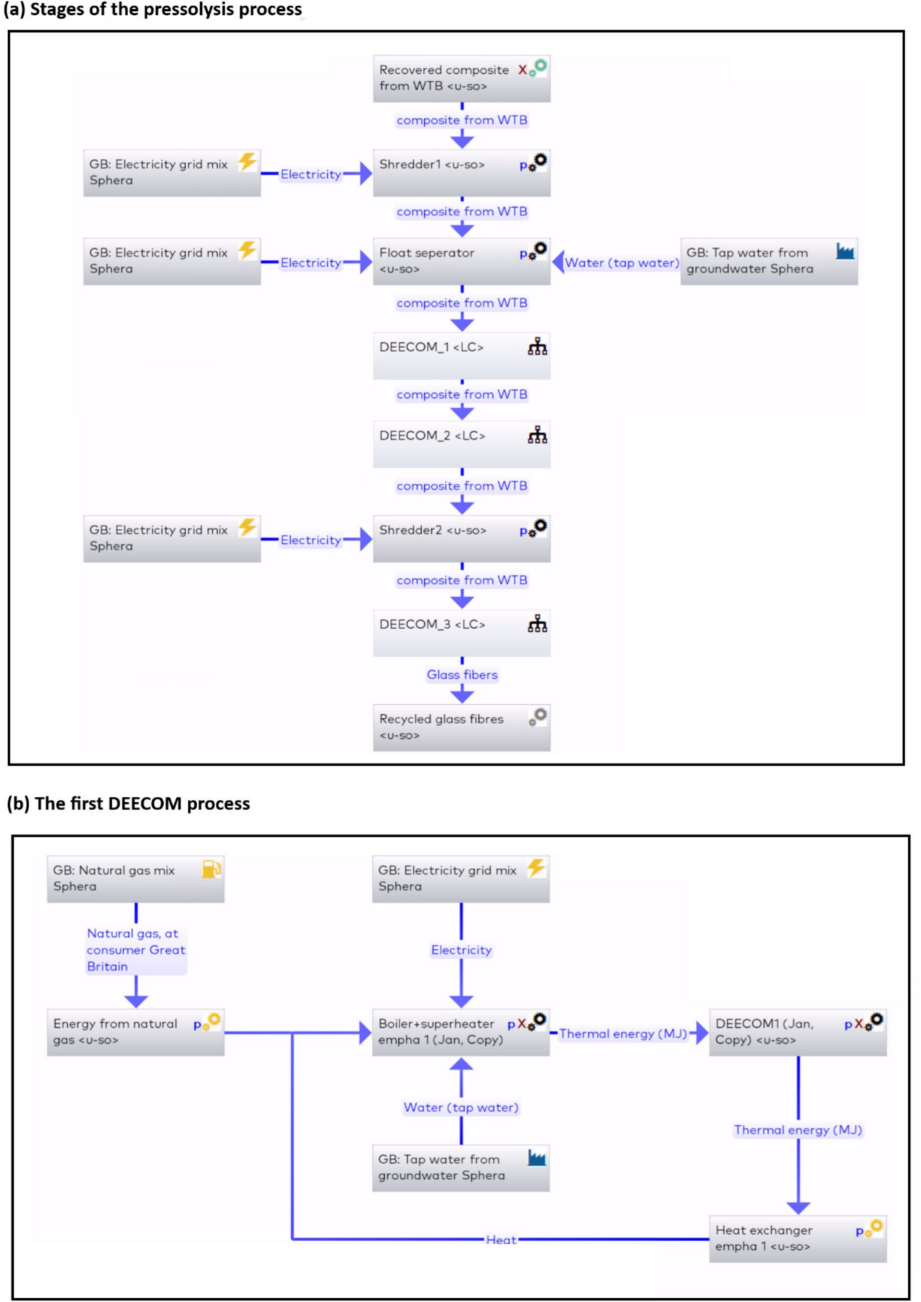
LCA model showing (a) the various stages of the pressolysis process and (b) the first DEECOM subprocess

The life cycle inventory is shown in Table 3. It shows the energy inputs provided to the various stages of the process. The process is modelled in *LCA for Experts* and analysed to compare its environmental impact with the batch process. The overall process and a representative subprocess (one of the pressolysis stages) are shown in Fig. 7.

**Table 3:** Life cycle inventory for the improved process (Scenario 2)

Stage	Item	Value
Initial input	Composite waste from wind turbine blades	100 kg
Shredder 1	Electricity	0.45 kWh
Float separator	Electricity	9.79 kWh
	Water	12,000 l
DEECOM chamber 1	Natural gas	40.12 m^3^
	Electricity	21 kWh
	Water	920 l
DEECOM chamber 2	Natural gas	38.88 m^3^
	Electricity	217 kWh
	Water	865 l
Shredder 2	Electricity	0.45 kWh
DEECOM chamber 3	Natural gas	49.95 m^3^
	Electricity	272 kWh
	Water	1352 l
Final output	Recovered fibres	33 kg

### *Scenario 3:* Improvement of the Process by Scale-Up

The benefits obtained from the modified process described in Sect.  3.2 can be further improved by scaling up the process. This involves determining the additional equipment necessary to achieve a larger scale of manufacturing and calculating the environmental impact that the scaling up imposes. 3 sub-scenarios 3a, 3b and 3c are considered and the details are described in this section.

#### Scenario 3a: Multiple Number of Runs

For Scenario 3a, we assume that the equipment available is the same as that used in Scenario 2. This places the constraint that all the components have a maximum material capacity of 100 kg and means that multiple runs of each component need to be performed to accommodate the larger input material at higher scales, leading to longer total run times. The number of runs required for the various scales considered is shown in Table 4.

**Table 4:** Number of runs of each component required at the various output scales (Scenario 3a and 3b)

Recovered fibre quantity (kg)	Scenario	Number of runs (Scenario 2a) or components (Scenario 2b) required					
		*Shredder 1*	*Float separator*	*Pressolysis chamber 1*	*Pressolysis chamber 1*	*Shredder 2*	*Pressolysis chamber 3*
1.29	1	-	-	1	-	-	-
33	2	1	1	1	1	1	1
66	3a and 3b	2	2	2	2	1	1
132		4	4	4	4	2	2
264		8	8	7	7	4	4

#### Scenario 3b: Multiple Number of Components

Next, we consider the scenario in which additional equipment can be obtained as per the requirement in each scale analysed but the maximum material capacity of each equipment is kept the same as that in Scenario 3a. For this case, the information in Table 4 is still relevant but the number of runs becomes the number of individual components required. If each component is considered to run independently of the others, the environmental impact is the same as Scenario 3a. There will be an increase in the initial cost incurred. However, the total run time is reduced since having multiple instances of each component means that they can be run simultaneously.

#### Scenario 3c: Increased Capacity of Components

The final scenario considers that, instead of increasing the number of runs or the number of individual components, it is the capacity of the components that is changed to accommodate the increase in material for larger scales. Also, a stepped increase in the capacity is assumed with steps of 100 kg. The necessary capacities of each component for this case are provided in Table 5.

**Table 5:** Capacity of each component required at the various output scales (Scenario 3c)

Recovered fibre quantity (kg)	Scenario	Capacity of each component (kg) (Scenario 3c)					
		*Shredder 1*	*Float separator*	*Pressolysis chamber 1*	*Pressolysis chamber 2*	*Shredder 2*	*Pressolysis chamber 3*
1.29	1	-	-	100	-	-	-
33	2	100	100	100	100	100	100
66	3c	200	200	200	200	100	100
132		400	400	400	400	200	200
264		800	800	700	700	400	400

## Results and Discussion

**Fig. 8:**
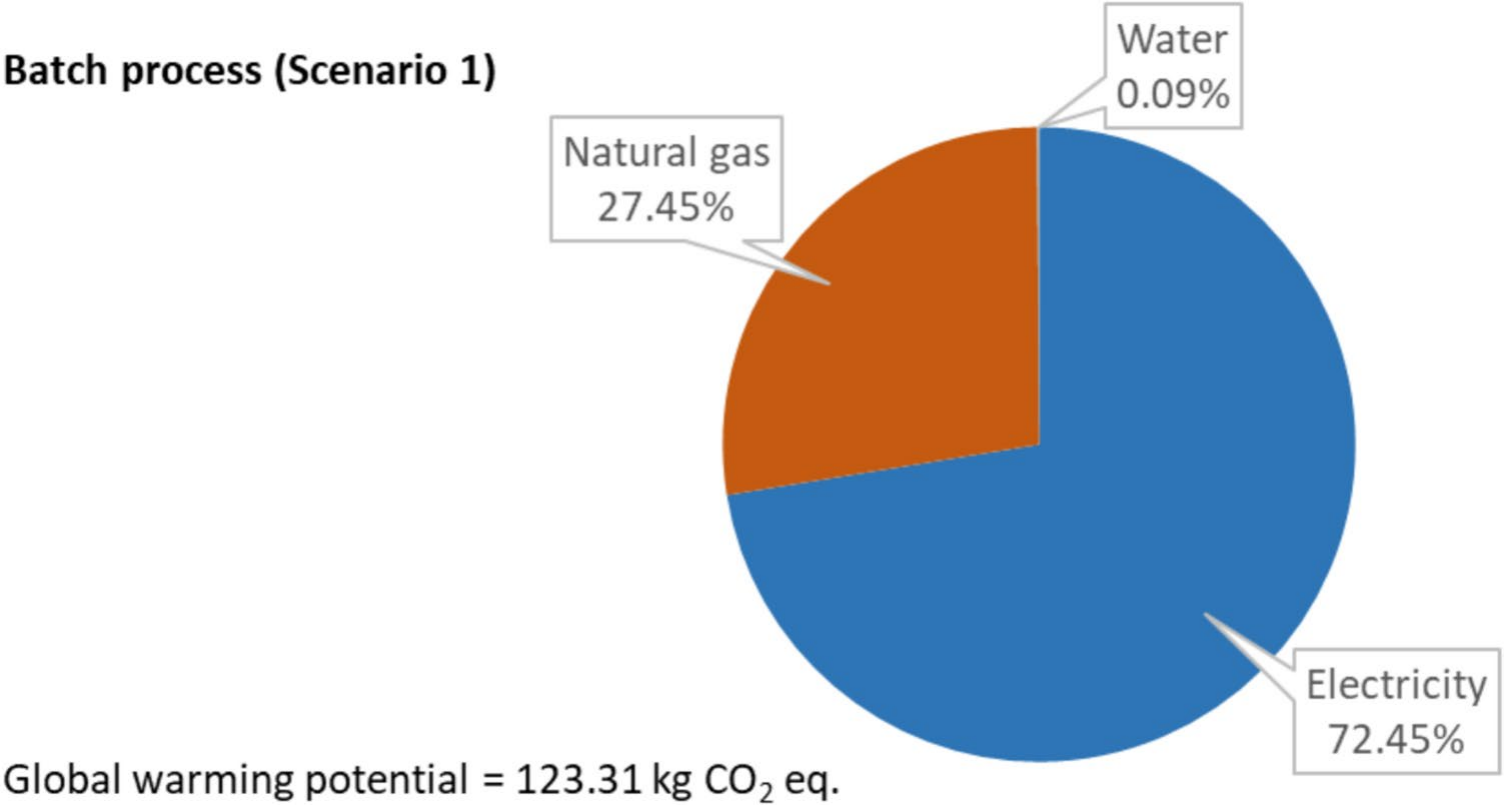
Climate change indicator value for the batch process (Scenario 1) and the split-up between different inputs

The climate change indicator used in this study is the global warming potential (GWP) taken from the ReCiPe 2016 methodology, which is a measure of the integrated infrared radiative forcing increase of greenhouse gases [28]. The GWP values for the process with various scales are shown in the figures below. Figure 8 shows the environmental impact indicator for the batch process (Scenario 1) and the split-up of the impact between the various energy inputs.

**Fig. 9:**
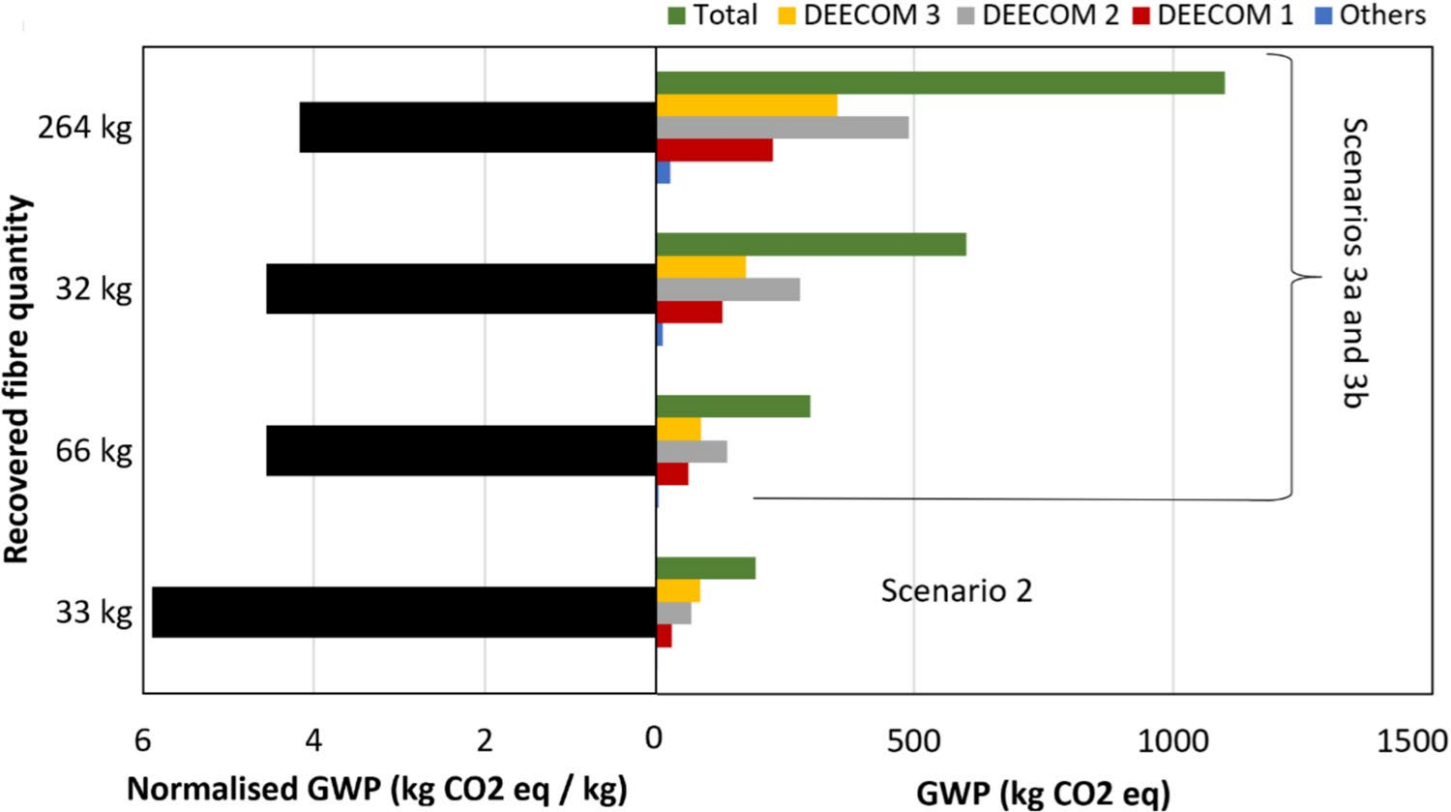
The climate change indicator (GWP) values for the various components and the entire process for Scenarios 2, 3a and 3b

**Fig. 10:**
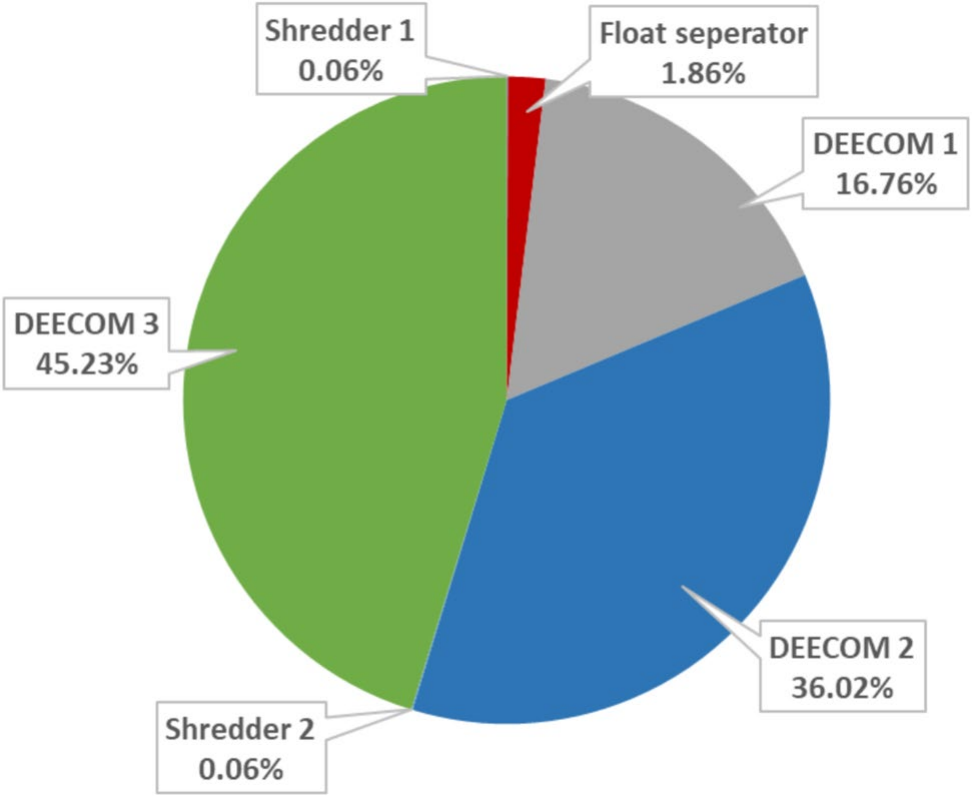
The split up of the climate change indicator of various components in Scenario 2a

**Fig. 11:**
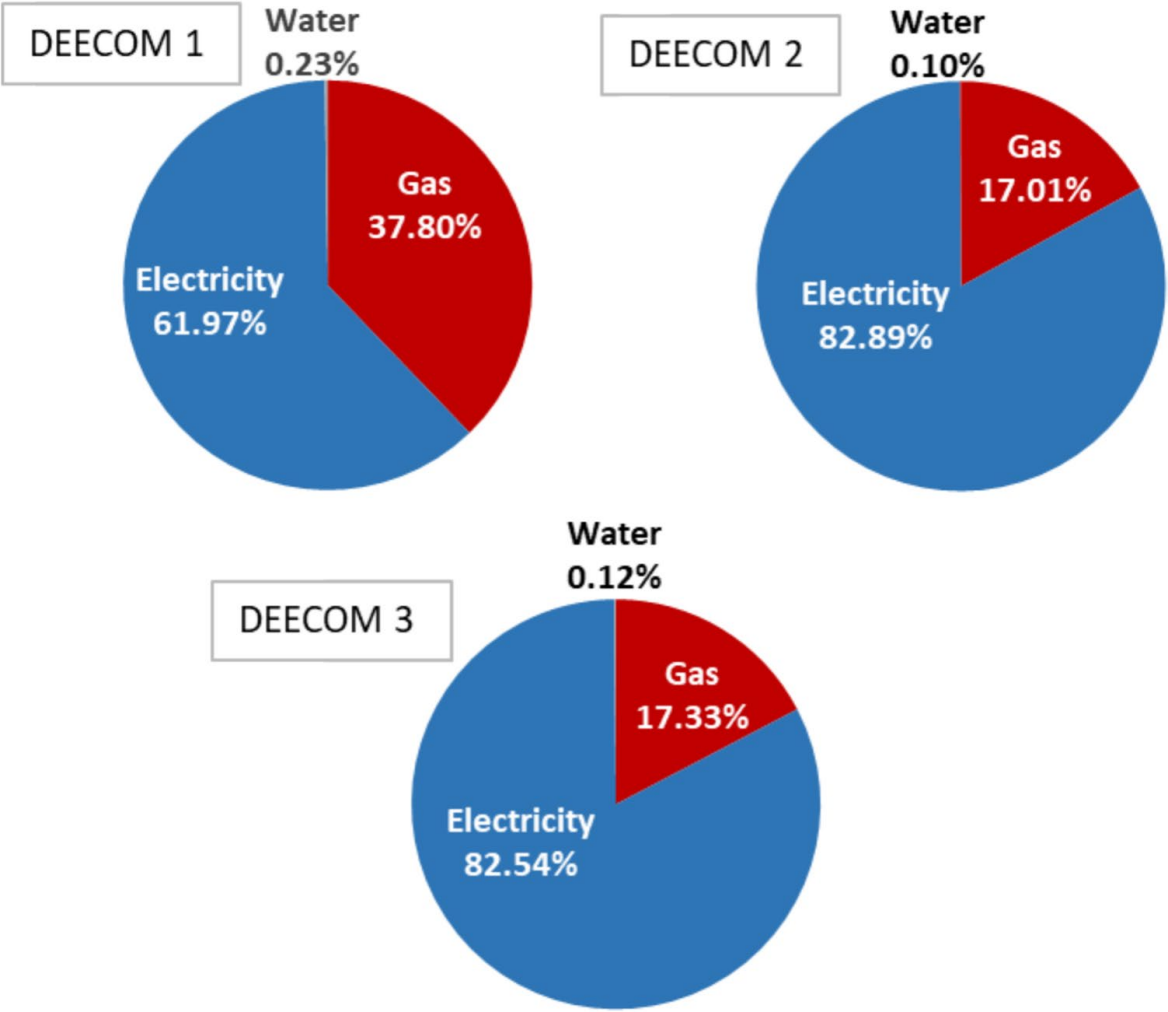
The split up of the climate change indicator in the three DEECOM stages in Scenario 2a

Figure 9 shows the GWP values for Scenario 2 compared with Scenarios 3a and 3b. There is an increase in the absolute value of the environmental impact indicator as the scale is increased due to the increase in the larger number of runs in Scenario 3a or the number of components in Scenario 3b. To enable easy calculation of the quantitative benefit of the modified process compared to the batch process and virgin glass fibre production, the GWP values have been normalised with respect to recovered fibre weight and are summarised in Table 6. The normalised GWP values with respect to per kilo weight of fibre recovered show that the scaling up has significant benefits. The normalised climate change indicator for the 264 kg scale of Scenarios 3a and 3b is nearly 30% lower than Scenario 2a (33 kg). Most of the global warming impact occurs in the pressolysis stages while the impact of the shredders and the float separator is relatively low. This is shown graphically in Fig. 10 which is the split-up of the total environmental impact between the various components in the process for Scenario 2. The split-up of Scenarios 3a, 3b and 3c also follow nearly the same pattern. Figure 11 shows the split up of the environmental impact of various energy inputs within the pressolysis stages in Scenario 2. The electricity mix stage causes most of the impact followed by the natural gas mix stage. The lower effect of natural gas is also due to the recovery of waste heat which reduces the consumption of natural gas in the boilers significantly.

**Table 6:** Comparison between the normalised environmental impact parameters of the scenarios in the current study and conventional processes (all values are normalised with respect to 1 kg of fibre recovered)

Process		Description	Recovered fibre quantity *kg*	Global warming potential (GWP) *kg CO*_*2*_* eq / kg fibre*
DEECOM Scenarios	Scenario 1	Batch process	1.29	95.59
	Scenario 2	Improved process with heat recovery	33	5.90
	Scenarios 3a and 3b	Scaled up process with multiple number of runs/components	66	4.56
			132	4.56
			264	4.17
	Scenario 3c	Scaled up process with increased capacity of components	66	3.77
			132	2.87
			264	2.07
Landfilling waste composite and producing virgin glass fibres				1.72
Reference fibre recovery methods^1^ [30]		Pyrolysis		1.52
		Solvolysis		1.92

^1^The figures reported are for the recovery of carbon fibre rather than glass fibre from waste composites consisting of a matrix that may be different from the study conducted. However, they are reported here for comparison of the current process with existing conventional fibre recovery methods

As mentioned earlier, Scenario 3c involves increasing the capacity of the individual components in the process as opposed to increasing either the number of runs (Scenario 3a) or the number of components itself (Scenario 3b). Scenario 3c is a conceptual exercise and the actual energy inputs required for the components with increased capacity have not been experimentally obtained. However, a scaling formula according to (Tribe and Alpine, 1986) is used to obtain the energy inputs (costs) for the scaled-up capacities of the various components. The formulation is given as

$$\frac{{C_{1} }}{{C_{2} }} = \left( {\frac{{S_{1} }}{{S_{2} }}} \right)^{\alpha }$$

where ${C}_{1}/{C}_{2}$ is the environmental impact ratio between the lower and higher scale processes, ${S}_{1}/{S}_{2}$ is the corresponding scale ratio, and $\alpha$ is the scale coefficient. A scale coefficient of 0.6 is used. The environmental impact values at the various scales of Scenario 3c are modelled in the software using the obtained energy inputs for the components.

**Fig. 12:**
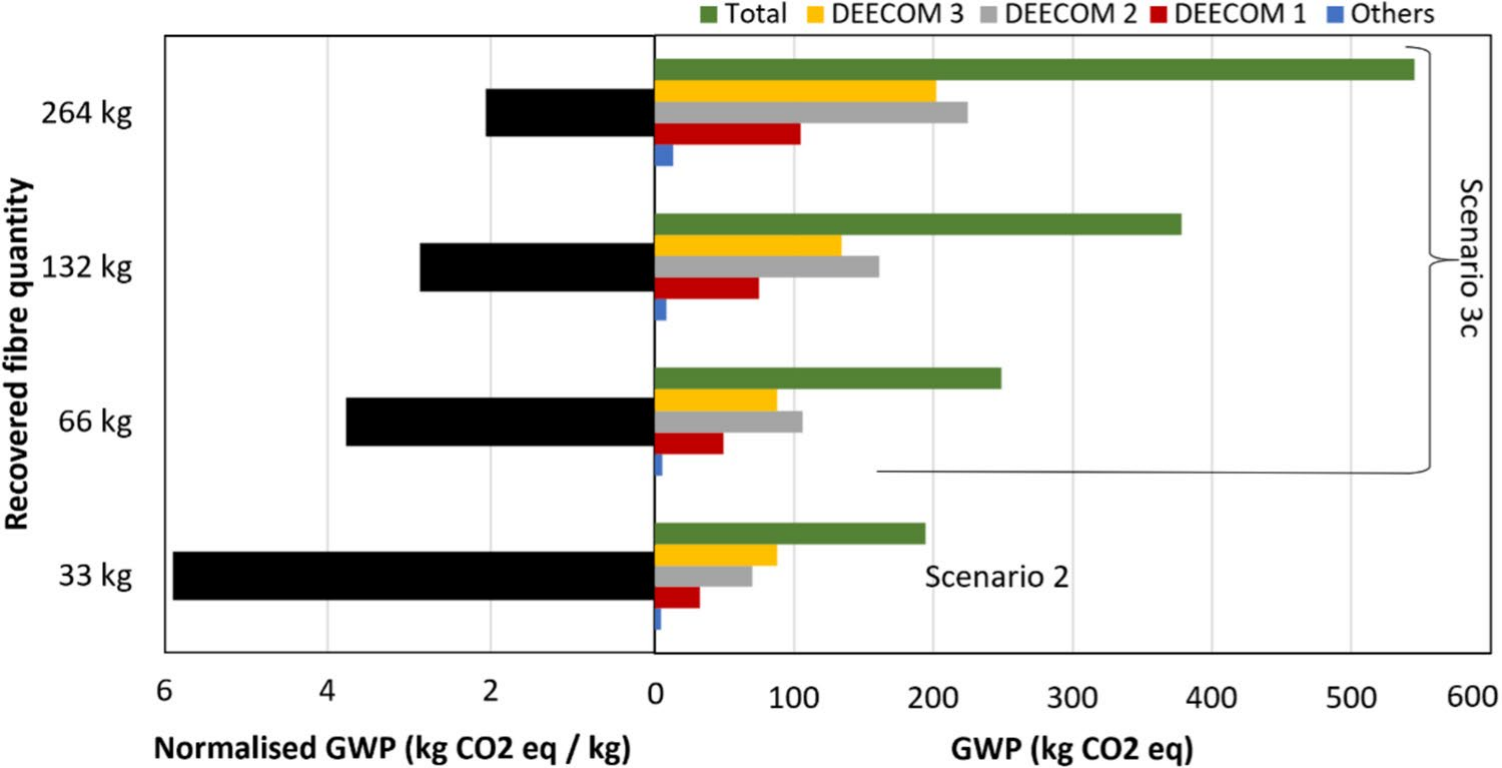
The climate change indicator values for the various components and the entire process for Scenarios 2 and 3c

Figure 12 compares the GWP values for Scenario 2 with the results of Scenario 3c. The normalised GWP values decrease substantially with increase in the scale of the process. When compared to Scenarios 3a and 3b, the results also show a significant decrease in the impact values for Scenario 3c. Compared to the cases where the number of runs or components is increased (Scenarios 3a and 3b), Scenario 3c where the capacity of the components is modified decreases the normalised GWP values from 4.17 kg CO_2_ eq / kg recovered fibre to 2.07 kg CO_2_ eq / kg recovered fibre for the 264 kg output scale case. This reduction is due to the fact that the environmental impact of operating the components in the process is not directly proportional to the size of a component. According to the scaling law used, doubling the size of any component increases the environmental impact by only ~ 1.5 times. Beyond this, using lower number of components which are larger in size also reduces the number of runs and thus reduces the number of times the individual components need to be pre-processed for each run, which is an additional benefit.

**Fig. 13:**
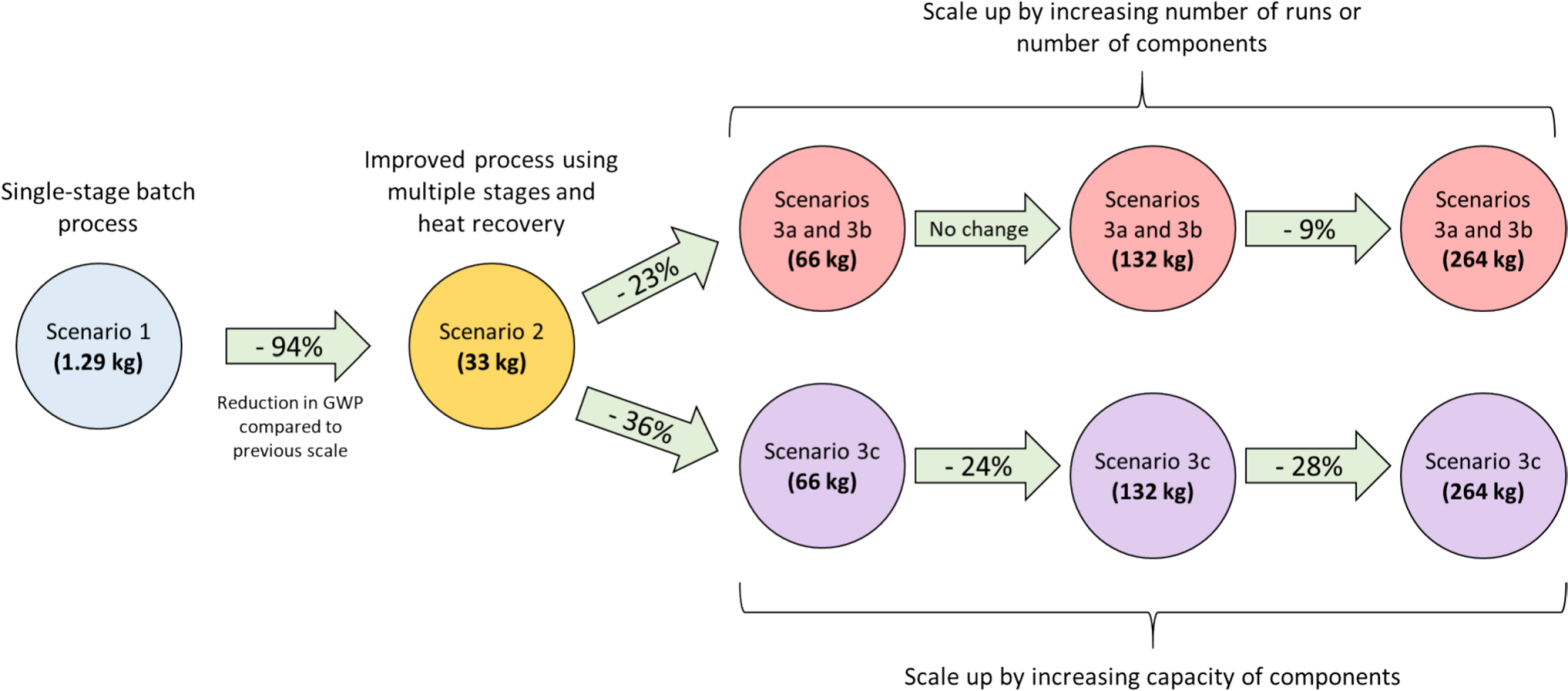
The various scenarios analysed and the reduction in GWP between subsequent output scales of the process

The percentage change of GWP values for each scale studied is tabulated in Table 7 and shown graphically in Fig. 13. As expected, scaling up from the batch process (Scenario 1) to Scenario 2a (33 kg recovered fibre) reduces the environmental impact significantly. However, scaling up from Scenario 2 by increasing the number of runs or components (Scenarios 3a and 3b) does not result in a reduction in GWP for every scale increase. For example, scaling up from the 66 kg to 132 kg recovered fibre output does not reduce the relative environmental impact. This is because the doubling of the output fibre quantity is achieved by doubling of the number of runs or components required, as seen in Table 4, and therefore this does not provide any benefit. This is not true between the 132 kg to 264 kg scale processes, and we again see a benefit since the number of runs or components required for the larger scale here is less than double of the lower scale, thus reducing the relative environmental impact. The maximum benefit of scaling up is seen in the case of increasing the component capacity instead of the number of runs or number of components (Scenario 3c).

**Table 7:** Change in the GWP values with increasing output scale

Reference scenario		Scaled-up scenario		% decrease in GWP
Scenario	Recovered fibre quantity *(kg)*	Scenario	Recovered fibre quantity *(kg)*	
1	1.29	2	33	94%
2	33	3a and 3b	66	23%
3a and 3b	66	3a and 3b	132	0%
3a and 3b	132	3a and 3b	264	9%
2	33	3a and 3b	66	36%
3a and 3b	66	3a and 3b	132	24%
3a and 3b	132	3a and 3b	264	28%

The environmental impact of the scenarios in the current study is compared with existing conventional end-of-life scenarios and material production methods. The GWP values for the landfilling process of waste composite parts and production of virgin glass fibres obtained from the *LCA for Experts* software is used since using pressolysis saves the composite from being disposed of in landfills and replaces the need for the production of new material. The normalised value of the GWP is 1.72 kg CO_2_ eq/kg fibre landfilled and produced (Table 6). For comparison, the case with the lowest environmental impact in the current study is the 264 kg output case of Scenario 3c with a normalised GWP of 2.07 kg CO_2_ eq/kg recovered fibre. Also, the greenhouse gas emission figures per kilogram for recovering carbon fibres from composites consisting of commonly used resin systems using conventional methods are also reported in Table 6. The CO_2_ equivalent emissions from pyrolysis were 1.52 kg CO_2_ eq/kg and that from solvolysis were 1.92 kg CO_2_ eq/kg [29]. With further improvements to the process, this value can be brought lower and comparable to the environmental impact of the conventional composite recycling and fibre production process. The potential of the scaled-up pressolysis process to make recycling of waste composites environment-friendly can be improved by recovering usable resin from the aqueous solution after the process in the chamber in addition to the recovery of the fibres. This will help by providing a higher level of circularity and decrease the environmental impact.

## Conclusion

Exploring efficient composite recycling technologies and scaling them to industrial levels is critical for achieving a sustainable and circular economy. This study demonstrates, through a life cycle assessment (LCA), that the pressolysis (DEECOM®) process can deliver substantial environmental benefits when optimised and scaled. The main findings are:

- Scaling up pressolysis from a small-scale batch system to a high-capacity process reduces the GWP from 95.6 kg CO₂ eq/kg fibre to 2.07 kg CO₂ eq/kg fibre, representing an overall ~ 98% improvement. This confirms that scale-up is essential to unlock the environmental advantages of the technology and bring it closer to industrial feasibility.
- The semi-continuous process with integrated heat recovery achieved a 94% reduction in GWP compared to the batch process. By recovering and reusing waste heat, natural gas consumption was reduced by ~ 60%, highlighting that process optimisation and energy recovery strategies are key enablers for sustainable large-scale operation.
- Among the different scaling approaches investigated (multiple runs, multiple units, and increased equipment capacity), enlarging component size (Scenario 3c) proved the most effective. This pathway further reduced GWP by 65% compared to the semi-continuous process, demonstrating that economies of scale are best realised by designing higher-capacity equipment rather than replicating smaller units.
- At scale, pressolysis approaches the environmental performance of pyrolysis (1.52 kg CO₂ eq/kg fibre) and solvolysis (1.92 kg CO₂ eq/kg fibre). Unlike these thermal and chemical routes, however, DEECOM® maintains fibre integrity, avoiding the degradation in strength and surface properties typically observed with pyrolysis and solvolysis. This makes pressolysis particularly promising for high-value fibre reuse.
- By enabling the recovery of intact fibres from end-of-life wind turbine blades and reducing dependency on virgin raw materials, pressolysis helps close the loop for thermoset composites. The reduced environmental footprint, combined with the avoidance of landfilling and incineration, positions DEECOM® as a pathway technology for achieving circular economy objectives in the composites sector.

Future work should focus on refining the process for industrial implementation, particularly by exploring resin recovery alongside fibre recovery to enhance material circularity. Additional emphasis should be placed on process energy optimisation, full-scale techno-economic analysis, and incorporating uncertainty analyses in future LCAs.

## Data Availability

The datasets generated during and/or analysed during the current study are not publicly available but are available from the corresponding author on reasonable request.

## Abbreviations

EoL: End of life
GWP: Global warming potential
LCA: Life cycle assessment
LCI: Life cycle inventory
SDG(s): Sustainable development goal(s)
UN: United nations
